# Use of platelet-rich fibrin for the treatment of periodontal intrabony defects: a systematic review and meta-analysis

**DOI:** 10.1007/s00784-021-03825-8

**Published:** 2021-02-20

**Authors:** Richard J. Miron, Vittorio Moraschini, Masako Fujioka-Kobayashi, Yufeng Zhang, Tomoyuki Kawase, Raluca Cosgarea, Soren Jepsen, Mark Bishara, Luigi Canullo, Yoshinori Shirakata, Reinhard Gruber, Döri Ferenc, Monica Diuana Calasans-Maia, Hom-Lay Wang, Anton Sculean

**Affiliations:** 1 Department of Periodontology, University of Bern, Bern, Switzerland; 2 Department of Periodontology, Dental Research Division, School of Dentistry, Veiga de Almeida University, Rio de Janeiro, Brazil; 3 Department of Cranio-Maxillofacial Surgery, Inselspital, Bern University Hospital, University of Bern, Bern, Switzerland; 4 Department of Oral Implantology, University of Wuhan, Wuhan, China; 5 Division of Oral Bioengineering, Institute of Medicine and Dentistry, Niigata University, Niigata, Japan; 6 Department of Prosthetic Dentistry, University Iuliu Hatieganu, Cluj-Napoca, Romania; 7 Department of Periodontology, Operative and Preventive Dentistry, University of Bonn, Bonn, Germany; 8 Division Private practice, West Bowmanville Family Dental, Bowmanville, Ontario Canada; 9 Independent researcher, Rome, Italy; 10 Department of Periodontology, Kagoshima University Graduate School of Medical and Dental Sciences, Kagoshima, Japan; 11 Department of Oral Biology, University of Vienna, Vienna, Austria; 12 Department of Periodontology, Semmelweis University, Budapest, Hungary; 13 Department of Oral Surgery, School of Dentistry, Fluminense Federal University, Rua Mario dos Santos Braga, 30, Centro, Niteroi, Rio de Janeiro, Brazil; 14 Department of Periodontics and Oral Medicine, University of Michigan, Ann Arbor, Michigan USA

**Keywords:** Intrabony defect, Platelet-rich fibrin, L-PRF, Advanced-PRF

## Abstract

**Objectives:**

This study aims to compare the treatment outcomes of periodontal intrabony defects by using platelet-rich fibrin (PRF) with other commonly utilized modalities.

**Materials and methods:**

The eligibility criteria comprised randomized controlled trials (RCTs) comparing the clinical outcomes of PRF with that of other modalities. Studies were classified into 10 categories as follows: (1) open flap debridement (OFD) alone versus OFD/PRF; (2) OFD/bone graft (OFD/BG) versus OFD/PRF; (3) OFD/BG versus OFD/BG/PRF; (4–6) OFD/barrier membrane (BM), OFD/PRP, or OFD/enamel matrix derivative (EMD) versus OFD/PRF; (7) OFD/EMD versus OFD/EMD/PRF; (8–10) OFD/PRF versus OFD/PRF/metformin, OFD/PRF/bisphosphonates, or OFD/PRF/statins. Weighted means and forest plots were calculated for probing depth (PD), clinical attachment level (CAL), and radiographic bone fill (RBF).

**Results:**

From 551 articles identified, 27 RCTs were included. The use of OFD/PRF statistically significantly reduced PD and improved CAL and RBF when compared to OFD. No clinically significant differences were reported when OFD/BG was compared to OFD/PRF. The addition of PRF to OFD/BG led to significant improvements in CAL and RBF. No differences were reported between any of the following groups (OFD/BM, OFD/PRP, and OFD/EMD) when compared to OFD/PRF. No improvements were also reported when PRF was added to OFD/EMD. The addition of all three of the following biomolecules (metformin, bisphosphonates, and statins) to OFD/PRF led to statistically significant improvements of PD, CAL, and RBF.

****Conclusions**:**

The use of PRF significantly improved clinical outcomes in intrabony defects when compared to OFD alone with similar levels being observed between OFD/BG and OFD/PRF. Future research geared toward better understanding potential ways to enhance the regenerative properties of PRF with various small biomolecules may prove valuable for future clinical applications. Future research investigating PRF at histological level is also needed.

**Clinical relevance:**

The use of PRF in conjunction with OFD statistically significantly improved PD, CAL, and RBF values, yielding to comparable outcomes to OFD/BG. The combination of PRF with bone grafts or small biomolecules may offer certain clinical advantages, thus warranting further investigations.

**Supplementary Information:**

The online version contains supplementary material available at 10.1007/s00784-021-03825-8.

## Introduction

Periodontal disease is one of the most prevalent chronic diseases known to man that begins as a superficial inflammatory response of the gingiva (gingivitis) and later progresses to attachment loss with subsequent destruction of the tooth-supporting structures (periodontitis) [1–4]. Results investigating the distribution of the disease from a national survey conducted in the USA found that over 47% of the adult population was affected with 38.5% of the population having either moderate or severe cases (stage III or stage IV) [5]. This finding is most alarming as the disease is characterized with an exponentially more difficult resolution and regeneration once advanced progression has taken place.

Treatment of periodontal disease is therefore of utmost importance since epidemic studies have linked periodontitis to a number of systemic diseases including cardiovascular diseases (heart attack/stroke), Alzheimer’s, diabetes, obesity, and premature births, among others [6]. It therefore becomes vital to correct the disease as early as possible and halt disease progression and utilize strategies to promote their regeneration [7–9].

True and complete periodontal regeneration is complex since it consists of a complex interaction of epithelium, gingival connective tissue, periodontal ligament, and alveolar bone [1]. True periodontal regeneration should also include Sharpey’s fibers spanning from the cementum through the periodontal ligament (PDL) and into the alveolar bundle bone [1]. To date, many attempts utilizing various strategies including bone grafts, barrier membranes, and biologic agents have been proposed, yet to date complete periodontal regeneration remains very challenging and unpredictable [1].

One strategy that was proposed several years ago for the regeneration of intrabony defects was the use of platelet concentrates [10]. While platelet-rich plasma (PRP) was proposed as a first-generation platelet concentrate, the use of anticoagulants has since been shown to interfere with the angiogenic and regenerative responses mediated by platelets [11]. For these reasons, a second-generation platelet concentrate, termed platelet-rich fibrin (PRF), has more been introduced in regenerative medicine and dentistry [10, 12–15].

Since PRF was first launched more than two decades ago in regenerative medicine, its use has gained widespread acceptance across many fields of medicine including for periodontal regeneration where nearly 40 randomized clinical trials (RCTs) have investigated its regenerative potential. One of the advantages of PRF is that following centrifugation, it forms a fibrin-dense clot with host platelets and leukocytes being entrapped favoring a more extended release of growth factors over time [16, 17]. A number of systematic reviews (SRs) have thoroughly documented the use of PRF in regenerative dentistry, where it has been shown to particularly favor soft tissue healing over hard tissue healing [10, 18, 19]. The aim of this systematic review with meta-analysis was to evaluate the current evidence regarding the use of PRF for the treatment of both intrabony defects in comparison to other treatment options including bone grafts, barrier membranes, enamel matrix derivative (EMD), and a number of other biomolecules commonly utilized for periodontal regeneration.

## Materials and methods

### Protocol

This SR followed the recommendations of the PRISMA guidelines [20]. The protocol for this SR was based on PRISMA-P [21]. There were no deviations from the initial protocol.

### Focused question

What is the effectiveness of PRF for the treatment of periodontal two- and three-walled intrabony defects?

### Eligibility criteria and study selection process

The inclusion criteria were based on the PICOS strategy highlighted below [22]. The search-and-screening process was conducted by two independent reviewing authors (R.J.M and V.M.), commencing with the analysis of titles and abstracts. Next, full papers were selected for careful reading and matched with the eligibility criteria for future data extraction. Disagreements between the reviewing authors were resolved through careful discussion. Only studies meeting the following criteria were included:

- Population: Systemically healthy humans with periodontal intrabony defects (two or three walls).
- Intervention: Surgical treatment of bone defects through the use of PRF alone or in combination with other biomaterials with a follow-up period of at least 6 months.
- Comparison: PRF versus open flap debridement (OFD) alone or in combination with other biomaterials.
- Outcomes: The outcome variable and data collection included the change in pocket depth (PD), clinical attachment level (CAL), and radiographic bone fill (RBF).
- Study design: RCTs with a minimum of 10 patients.

### Search strategy

PubMed/MEDLINE, the Cochrane Central Register of Controlled Trials, Scopus, Embase, and Lilacs were used to search for articles that were published before June 2020 without other restrictions regarding date or language. A search of the gray literature using the Literature Report [23] and OpenGrey [24] databases was also conducted. Finally, the study reference lists were evaluated (cross-referenced) to identify other studies for potential inclusion. The search strategy is described in the Supplementary Appendix (S1). Retrospective clinical studies, case reports, or animal studies as well as follow-up of less than 6 months were excluded from the study.

### Data synthesis

The study data were extracted by R.J.M. and M.F.K. and systematically reviewed by V.M. The following data, when available, were extracted from the included studies: authors, study design, follow-up, number of treated intrabony defects, type of bone defects, number of subjects, age range, gender, number of smokers, surgical technique, mean difference (mD) in PD, CAL, BF, centrifugation system, volume of blood drawn, and centrifugation parameters.

### Assessments of the risk of bias

Two reviewing authors (V.M. and M.D.C.M.) analyzed the risk of bias. The RoB 2 (a revised Cochrane risk-of-bias tool for randomized trials) [25] was used to analyze the risk of bias in RCTs. Each study was analyzed in relation to five domains: risk of bias arising from the randomization process, risk of bias due to deviations from the intended interventions, missing outcome data, risk of bias in the measurement of the outcome, and risk of bias in the selection of the reported research. Studies were classified as having a low risk, some concerns, or high risks of bias for each domain. The overall risk of biased judgment used the following criteria: low risk, when the five areas of the study were judged as low risk; some concerns, when the study is judged as raising some concerns in at least one area; and high risk, when the study is judged to be at high risk in at least one domain or when the study is judged to have some concerns for multiple domains in a way that substantially lowers confidence in the result.

### Statistical analysis

The continuous variables (PD, CAL, and BF) of the included studies were categorized in groups and subgroups and analyzed in a meta-analysis through software Review Manager (version 5.2.8, Copenhagen, Denmark, 2014).

The estimates of the intervention effects were expressed as percentages or millimeters with 95% CIs. The inverse variance method was used for the random-effects or fixed-effects models, depending on the heterogeneity between the studies. The heterogeneity was assessed using *χ*^2^ tests. Values ≤ 25% were validated as low heterogeneity, while values > 25 < 50% was classified as moderate. Values ≥ 50% were classified as high heterogeneity [26]. The use of the random-effects model was conducted when heterogeneity was found (*p* < 0.10). In contrast, the fixed-effects model was used in the case of low or medium heterogeneity. The statistical significance level of the effect of meta-analysis was fixed in *p* < 0.05.

## Results

### Literature search

**Fig. 1:**
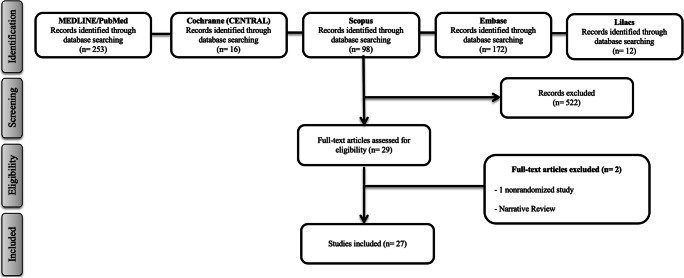
Flow diagram (PRISMA format) of the screening and selection process

The process of the search and selection and the reasons for excluding potential studies are shown in Fig. 1. Twenty-seven studies on intrabony defects [27–53] published between 2011 and 2019 met the eligibility criteria and were included in this SR. Of the 27 RCTs, the most highly researched centrifugation system utilized in 15 of 27 studies (56% of studies) was the Remi centrifuge whereas the IntraSpin/Hettich PC-02 system was utilized in 1/27 studies (4% of studies). Of the 27 studies, 20/27 utilized 3000 rpm for 10-min protocol (74% of studies) whereas 2/27 studies utilized 2700 rpm for 12-min protocol (7% of studies). Only 2 of 27 studies included smokers into their study.

### Study characteristics

The included studies analyzed 1025 research participants. In addition to OFD alone, the effect of PRF was compared to other groups of biomaterials (autograft, allograft, xenograft, alloplast, barrier membrane, enamel matrix derivative (EMD), metformin, bisphosphonates, and statins). The mean follow-up period of the studies was 8.44 ± 2.04 months. The data extracted from each included study are presented in Table 1.

**Table 1:** Main characteristics of the 27 RCTs included in the present study

**Authors (year)**	**Study design** **Follow-up**	**Number of participants** **Gender** **Mean age**	**Groups**	**Bone defect type**	**Smokers (no, yes)**	**Conclusions**
*OFD vs. PRF*						
Sharma and Pradeep (2011) [48]	RCT (parallel) 9 months	42 ♂24/♀18 35.3	C: 28, OFD T: 28, OFD + PRF	3 walls	No	There was greater PD reduction, CAL gain, and bone fill at sites treated with PRF with OFD compared to OFD alone
Thorat et al. (2011) [50]	RCT (parallel) 9 months	32 ♂20/♀12 30.7	C: 16, OFD T: 16, OFD + PRF	2 and 3 walls	No	There was greater reduction in PD, more CAL gain, and greater intrabony defect fill at sites treated with PRF than the OFD alone
Rosamma et al. (2012) [53]	CT (split-mouth) 12 months	15 ♂6/♀9 29.5	C: 15, OFD T: 15, OFD + PRF	2 and 3 walls	No	The use of PRF was more effective than OFD alone in the management of IBDs
Ajwani et al. (2015) [28]	RCT (split-mouth) 9 months	20 ♂10/♀10 30.5	C: 20, OFD T: 20, OFD + PRF	2 and 3 walls	No	Adjunctive use of PRF with OFD significantly improves defect fill when compared to OFD alone
Bajaj et al. (2017) [30]	RCT (parallel) 9 months	17 ♂9/♀8 29.7	C: 27, OFD T: 27, OFD + PRF	2 and 3 walls	No	There was greater BF at sites treated with PRF with conventional OFD than conventional OFD alone
Patel et al. (2017) [41]	RCT (split-mouth) 12 months	13 ♂4/♀9 44	C: 13, OFD T: 13, OFD + PRF	2 and 3 walls	No	The adjunctive use of PRF to conventional OFD may be potentially used in the treatment of IBDs
Pradeep et al. (2017) [42]	RCT (parallel) 9 months	62 ♂34/♀28 39.7	C: 18, OFD T1: 19, OFD + PRF T2: 20, OFD + PRF + HA	3 walls	No	Treatment of IBD with PRF results in significant improvements of clinical parameters compared to baseline
Thorat et al. (2017) [49]	RCT (split-mouth) 12 months	15 ♂7/♀8 25	C: 15, OFD T: 15, OFD + PRF	3 walls	NR	Use of PRF significantly enhances the clinical and radiographic outcomes of OFD in the treatment of IBDs
*BG vs. PRF*						
Mathur et al. (2015) [38]	RCT (parallel) 6 months	25 ♂14/♀11 39.7	C: 19, OFD + ABG T: 19, OFD + PRF	2 and 3 walls	No	The use of either PRF or ABG was effective in the treatment of IBDs
Shah et al. (2015) [47]	RCT (split-mouth) 6 months	20 NR NR	C: 20, OFD + DFDBA T: 20, OFD + PRF	2 and 3 walls	No	PRF has shown significant results after 6 months, which is comparable to DFDBA for periodontal regeneration
Chadwick et al. (2016) [32]	RCT (parallel) 6 months	36 ♂20/♀16 54.9	C: 19, OFD + DFDBA T: 17, OFD + PRF	2 and 3 walls	Yes	Treatment of IBDs with either DFDBA or PRF resulted in a significant gain in CAL as well as BF after 6 months of healing, with no significant difference
Galav et al. (2016) [34]	RCT (split-mouth) 9 months	20 NR 45	C: 20, OFD + ABG T: 20, OFD + PRF	2 and 3 walls	No	Both ABG and PRF can be used predictably to reconstruct lost periodontal structures
Yajamanya et al. (2017) [51]	RCT (parallel) 9 months	32 NR NR	C: 28, OFD T1: 28, OFD + BioG T2: 28, OFD + PRF	2 and 3 walls	No	This study shows marked improvements in the clinical parameters and radiographic outcomes with both BioG and PRF to treat periodontal IBDs as compared to OFD alone
*BG vs. BG + PRF*						
Bansal and Bharti (2013) [52]	RCT (split-mouth) 6 months	10 NR NR	C: 10, OFD + DFDBA T: 10, OFD + DFDBA + PRF	NR	NR	There was a significantly greater PD reduction and CAL when PRF was added to DFDBA
Elgendy and Abo Shady (2015) [33]	RCT (split-mouth) 6 months	20 NR 44	C: 20, OFD + HA T: 20, OFD + HA + PRF	NR	Yes	Both treatment groups showed a significant PD reduction and CAL gain 6 months after surgery. However, there was a significantly greater PD reduction and CAL gain when PRF was added to BG
Agarwal et al. (2016) [27]	RCT (split-mouth) 12 months	30 ♂15/♀15 52	C: 30, OFD + DFDBA T: 30, OFD + DFDBA/PRF	2 and 3 walls	No	The combination of PRF and DFDBA is more effective than DFDBA alone
Naqvi et al. (2017) [39]	RCT (split-mouth) 9 months	10 ♂7/♀3 NR	C: 10, OFD + BioG T: 10, OFD + BioG + PRF	2 and 3 walls	No	The results of this study showed both groups BioG putty alone and the combination of PRF and BioG putty are effective in the treatment of IBDs
Sezgin et al. (2017) [46]	RCT (split-mouth) 6 months	15 ♂8/♀7 NR	C: 15, OFD + ABBM T: 15, OFD + ABBM + PRF	2 and 3 walls	No	The results of this study indicate that both therapies are effective in the treatment of intrabony defects
Bodhare et al. (2019) [31]	RCT (split-mouth) 6 months	20 ♂11/♀9 35.9	C: 20, OFD + BioGide T: 20, OFD + BioGide + PRF	2 and 3 walls	No	BioG when used in combination with PRF is found to be more effective in gain in CAL, reduction in PD, and achieving greater bone fill as compared to treatment with BG alone
*BM vs. PRF*						
Panda et al. (2016) [40]	RCT (split-mouth) 9 months	18 ♂10/♀8 38.1	C: 18, OFD + BM T: 18, OFD + BM + PRF	3 walls	No	The adjunctive use of PRF in combination with BM is more effective in the treatment of IBDs in chronic periodontitis as compared with BM alone
*PRP vs. PRF*						
Pradeep et al. (2012) [45]	RCT (parallel) 9 months	54 ♂27/♀27 36.8	C: 17, OFD T1: 17, OFD + PRP T2: 16, OFD + PRF	3 walls	No	There was similar PD reduction, CAL gain, and BF at sites treated with PRF or PRP with conventional OFD
*EMD vs. PRF*						
Gupta et al. (2014) [35]	RCT (parallel) 6 months	30 ♂15/♀15 NR	C: 22, OFD + EMD T: 22, OFD + PRF	3 walls	No	Both EMD and PRF were effective in the regeneration of IBDs
*EMD vs. EMD + PRF*						
Aydemir Turkal et al. (2016) [29]	RCT (split-mouth) 6 months	28 ♂14/♀14 38.5	C: 24, OFD + EMD T: 25, OFD + EMD + PRF	1, 2, and 3 walls	No	Addition of PRF did not improve the clinical and radiographic outcomes
*PRF vs. PRF + metformin*						
Pradeep et al. (2015) [44]	RCT (parallel) 9 months	120 ♂60/♀60 41	C: 30, OFD T1: 30, OFD + 1% MF T2: 30, OFD + PRF T3: 30, OFD + 1% MF + PRF	3 walls	No	The study showed that the PRF + 1% MF group was more effective than MF, PRF, or OFD alone in the management of IBDs
*PRF vs. PRF + bisphosphonates*						
Kanoriya et al. 2016 [36]	RCT (parallel) 9 months	90 ♂43/♀47 40.3	C: 30, OFD T1: 30, OFD + PRF T2: 30, OFD + PRF/1% ALN	3 walls	No	Combined approach therapy of PRF + 1% ALN for IBD treatment showed better clinical parameter outcomes compared with PRF and OFD alone
*PRF vs. PRF + statins*						
Martande et al. (2016) [37]	RCT (parallel) 9 months	96 ♂48/♀48 37.6	C: 30, OFD T1: 30, OFD + PRF T2: 30, OFD + PRF + 1.2% ATV	3 walls	No	PRF + 1.2% ATV showed similar improvements in clinical parameters with a greater percentage radiographic defect depth reduction compared with PRF alone in treatment of IBDs
Pradeep et al. (2016) [43]	RCT (parallel) 9 months	90 ♂45/♀45 35	C: 30, OFD T1: 30, OFD + PRF T2: 30, OFD + PRF + 1.2% RSV	2 and 3 walls	No	OFD with RSV (1.2%) and PRF results in significantly greater periodontal benefits compared with OFD alone or with PRF
				**Methods for PRF preparation**		
**Authors (year)**	**Mean difference in PD between baseline and final follow-up (mm)**	**Mean difference in CAL between baseline and final follow-up (mm)**	**Mean difference in BF between** **baseline and final follow-up (mm)**	**Centrifugation system**	**Volume of blood drawn (ml)**	**Centrifugation parameters** **speed (rpm) × time (min)**
*OFD vs. PRF*						
Sharma and Pradeep (2011) [48]	3.21 ± 1.64 (C) 4.55 ± 1.87 (T)	2.77 ± 1.44 (C) 3.31 ± 1.76 (T)	0.09 ± 0.11 (C) 2.50 ± 0.78 (T)	R-4C (REMI, Mumbai, India)	10	3000 × 10
Thorat et al. (2011) [50]	3.56 ± 1.09 (C) 4.69 ± 1.45 (T)	2.13 ± 1.71 (C) 4.13 ± 1.63 (T)	1.24 ± 0.69 (C) 2.12 ± 0.69 (T)	NR	10	2700 × 12
Rosamma et al. (2012)	2.40 ± 0.63 (C) 4.67 ± 0.90 (T)	1.40 ± 1.06 (C) 4.73 ± 0.88 (T)	0.64 ± 0.50 (C) 1.93 ± 1.07 (T)	KW-70 (Almicro Instruments, Haryana, India)	10	3000 × 10
Ajwani et al. (2015) [28]	1.60 ± 0.84 (C) 1.90 ± 0.74 (T)	1.30 ± 0.68 (C) 1.80 ± 0.63 (T)	0.80 ± 0.35 (C) 1.45 ± 0.50 (T)	R-4C (REMI, Mumbai, India)	10	3000 × 10
Bajaj et al. (2017) [30]	2.14 ± 1.26 (C) 3.14 ± 1.26 (T)	1.59 ± 1.01 (C) 2.66 ± 1.07 (T)	0.84 ± 0.99 (C) 2.24 ± 0.66 (T)	R-4C (REMI, Mumbai, India)	10	3000 × 10
Patel et al. (2017) [41]	2.40 ± 0.84 (C) 4.20 ± 1.69 (T)	2.10 ± 0.74 (C) 3.70 ± 0.67 (T)	NR	REMI-8C (REMI, Mumbai, India)	10	3000 × 10
Pradeep et al. (2017) [42]	2.97 ± 0.93 (C) 3.90 ± 1.09 (T1) 4.27 ± 0.98 (T2)	2.67 ± 1.09 (C) 3.03 ± 1.16 (T1) 3.67 ± 1.03 (T2	0.93 ± 0.83 (C) 3.20 ± 0.89 (T1) 3.87 ± 1.33 (T2)	R-4C (REMI, Mumbai, India)	10	3000 × 10
Thorat et al. (2017) [49]	1.50 ± 0.34 (C) 4.00 ± 0.63 (T)	0.33 ± 1.21 (C) 4.00 ± 0.63 (T)	1.67 ± 0.06 (C) 3.09 ± 0.50 (T)	R-4C (REMI, Mumbai, India)	10	3000 × 12
*BG vs. PRF*						
Mathur et al. (2015) [38]	2.40 ± 1.06 (C) 2.67 ± 1.29 (T)	2.67 ± 1.63 (C) 2.53 ± 1.06 (T)	2.66 ± 1.84 (C) 2.93 ± 1.79 (T)	R-4C (REMI, Mumbai, India)	NR	3000 × 10
Shah et al. (2015) [47]	3.70 ± 0.68 (C) 3.67 ± 0.69 (T)	2.97 ± 1.68 (C) 2.97 ± 1.56 (T)	0.32 ± 1.59 (C) 0.42 ± 1.38 (T)	NR	10	3000 × 10
Chadwick et al. (2016) [32]	2.00 ± 1.37 (C) 2.12 ± 1.41 (T)	1.16 ± 1.33 (C) 1.03 ± 0.86 (T)	1.53 ± 1.64 (C) 1.35 ± 1.60 (T)	Centrifuge 1310 (Salvin Dental Specialties, Charlotte, NC)	10	3000 × 10
Galav et al. (2016) [34]	4.80 ± 0.57 (C) 4.10 ± 0.63 (T)	4.50 ± 0.52 (C) 3.90 ± 0.37 (T)	4.10 ± 0.47 (C) 4.59 ± 0.70 (T)	NR	10	3000 × 10
Yajamanya et al. (2017) [51]	3.68 ± 0.72 (C) 5.57 ± 1.10 (T1) 6.11 ± 0.92 (T2)	4.14 ± 0.76 (C) 6.57 ± 1.45 (T1) 6.74 ± 1.55 (T2)	NR	NR	10	3000 × 10
*BG vs. BG + PRF*						
Bansal and Bharti (2013) [52]	3.10 ± 0.74 (C) 4.00 ± 0.82 (T)	2.30 ± 0.70 (C) 3.40 ± 0.60 (T)	1.93 ± 1.21 (C) 2.13 ± 1.28 (T)	NR	10	3000 × 10
Elgendy and Abo Shady (2015) [33]	3.33 ± 0.36 (C) 3.30 ± 0.18 (T)	3.55 ± 0.13 (C) 3.50 ± 0.06 (T)	NR	NR	10	3000 × 10
Agarwal et al. (2016) [27]	3.60 ± 0.51 (C) 4.15 ± 0.84 (T)	2.61 ± 0.68 (C) 3.73 ± 0.74 (T)	2.49 ± 0.64 (C) 3.50 ± 0.67 (T)	NR	10	400×*g* × 12
Naqvi et al. (2017) [39]	3.15 ± 1.06 (C) 3.20 ± 2.30 (T)	3.15 ± 1.06 (C) 4.10 ± 1.73 (T)	5.70 ± 1.37 (C) 7.10 ± 1.37 (T)	NR	10	3000 × 10
Sezgin et al. (2017) [46]	4.21 ± 1.21 (C) 4.93 ± 1.22 (T)	3.27 ± 1.34 (C) 4.47 ± 1.60 (T)	1.98 ± 0.80 (C) 2.55 ± 1.15 (T)	PC-02 (Process, Nice, France)	10	2700 × 12
Bodhare et al. (2019) [31]	5.65 ± 1.66 (C) 5.75 ± 1.16 (T)	4.20 ± 1.70 (C) 5.05 ± 1.09 (T)	2.56 ± 0.95 (C) 3.51 ± 1.17 (T)	NR (REMI, Mumbai, India)	10	3000 × 10
*BM vs. PRF*						
Panda et al. (2016) [40]	3.19 ± 1.33 (C) 3.88 ± 1.15 (T)	3.38 ± 1.45 (C) 4.44 ± 1.50 (T)	0.80 ± 0.28 (C) 2.10 ± 0.64 (T)	Model C-854/6 (REMI, Mumbai, India)	5	3000 × 10
*PRP vs. PRF*						
Pradeep et al. (2012) [45]	2.97 ± 0.93 (C) 3.77 ± 1.07 (T1) 3.77 ± 1.19 (T2)	2.83 ± 0.91 (C) 2.93 ± 1.08 (T1) 3.17 ± 1.29 (T2)	0.13 ± 1.46 (C) 2.70 ± 0.79 (T1) 2.80 ± 0.89 (T2)	R-4C (REMI, Mumbai, India)	10	3000 × 10
*EMD vs. PRF*						
Gupta et al. (2014) [35]	1.80 ± 0.56 (C) 1.80 ± 0.77 (T)	2.00 ± 0.54 (C) 1.87 ± 0.91 (T)	2.08 ± 0.78 (C) 1.67 ± 1.17 (T)	NR (REMI, Mumbai, India)	10	3000 × 12
*EMD vs. EMD + PRF*						
Aydemir Turkal et al. (2016) [29]	3.88 ± 1.26 (C) 4.00 ± 1.38 (T)	3.29 ± 1.30 (C) 3.42 ± 1.28 (T)	1.21 ± 1.24 (C) 1.13 ± 0.83 (T)	Mikro 22 R (Hettich, Tuttlingen, Germany)	10	400×*g* × 10
*Metformin vs. PRF*						
Pradeep et al. (2015) [44]	3.00 ± 0.18 (C) 3.93 ± 0.25 (T1) 4.00 ± 0.18 (T2) 4.90 ± 0.30 (T3)	2.96 ± 0.18 (C) 3.93 ± 0.25 (T1) 4.03 ± 0.18 (T2) 4.90 ± 0.30 (T3)	0.49 ± 0.27 (C) 2.56 ± 0.28 (T1) 2.53 ± 0.30 (T2) 2.77 ± 0.30 (T3)	R-4C (REMI, Mumbai, India)	10	3000 × 10
*Bisphosphonates vs. PRF*						
Kanoriya et al. 2016 [36]	2.86 ± 0.68 (C) 3.70 ± 0.91 (T1) 4.53 ± 0.81 (T2)	3.03 ± 0.18 (C) 4.20 ± 0.66 (T1) 5.16 ± 0.46 (T2)	0.38 ± 0.26 (C) 2.42 ± 0.21 (T1) 2.84 ± 0.26 (T2)	R-4C (REMI, Mumbai, India)	10	3000 × 10
*Statins vs. PRF*						
Martande et al. (2016) [37]	2.76 ± 1.43 (C) 3.76 ± 1.12 (T1) 4.06 ± 1.22 (T2)	2.50 ± 1.33 (C) 3.40 ± 1.13 (T1) 3.66 ± 1.42 (T2)	0.27 ± 0.19 (C) 2.46 ± 0.33 (T1) 2.58 ± 0.36 (T2)	R-4C (REMI, Mumbai, India)	5	3000 × 12 to 14
Pradeep et al. (2016) [43]	3.10 ± 0.30 (C) 4.03 ± 0.18 (T1) 4.90 ± 0.31 (T2)	2.47 ± 0.77 (C) 3.30 ± 0.65 (T1) 3.93 ± 0.78 (T2)	1.43 ± 0.50 (C) 3.17 ± 0.65 (T1) 3.63 ± 0.67 (T2)	R-4C (REMI, Mumbai, India)	10	3000 × 10

RCT, randomized clinical trial; OFD, open flap debridement; IBDs, infra-bony defects; NR, not reported; C, control group; T, test group, ♂, male; ♀, female; PRF, platelet-rich fibrin; PRP, platelet-rich plasma; PD, probing depth; CAL, clinical attachment level; BF, bone fill; rpm, rotations per minute; BG, bone graft; HA, hydroxyapatite; DFDBA, demineralized freeze-dried bone allograft; XB, xenogeneic bone; ABBM, anorganic bovine bone mineral; ABG, autogenous bone graft; EMD, Emdogain; BioG, bioactive glass; BM, barrier membrane; MF, metformin; ALN, alendronate; ATV, atorvastatin; RSV, rosuvastatin

### Intrabony defects

#### Probing depth

**Fig. 2:**
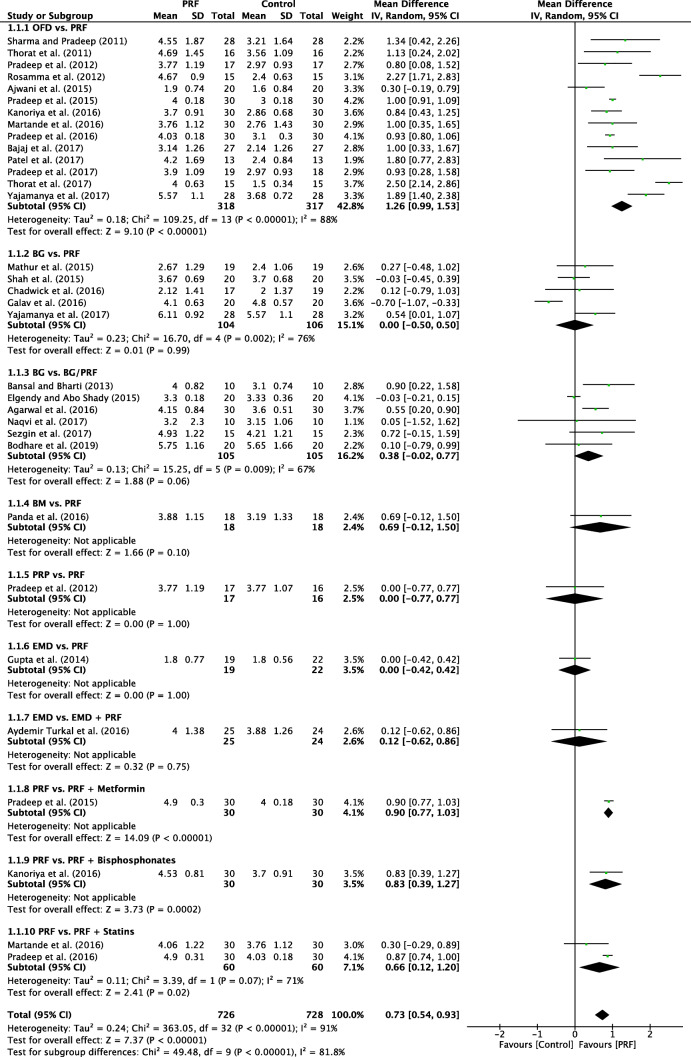
Forest plot for the event reduction in “probing depth” (PD) (reported in mm) for intrabony defects

**Fig. 3:**
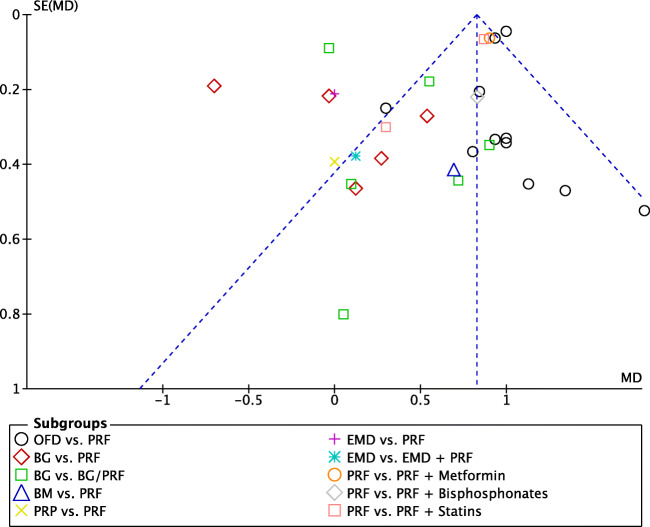
Funnel plot for the studies reporting reduction in probing depth (PD) (reported in mm) for intrabony defects

A random-effects model was used to evaluate the PD due to the high heterogeneity that was found between the subgroups (*P* < 0.00001; *I*^2^ = 91%). No subgroup showed a significant result in favor of the control groups when compared to that of PRF. In overall effect, the use of PRF differed significantly (*P* < 0.00001) in favor of PRF when compared with the control groups, with a mD of 0.82 (95% CI 0.78 to 0.87) (Fig. 2). The funnel plot demonstrated asymmetric distribution indicating high risk of publication bias (Fig. 3). The sensitivity analysis (exclusion of outliners) suggests that the divergence between the size of the sample groups may favor the increase in the possibility of publication bias.

#### Clinical attachment level

**Fig. 4:**
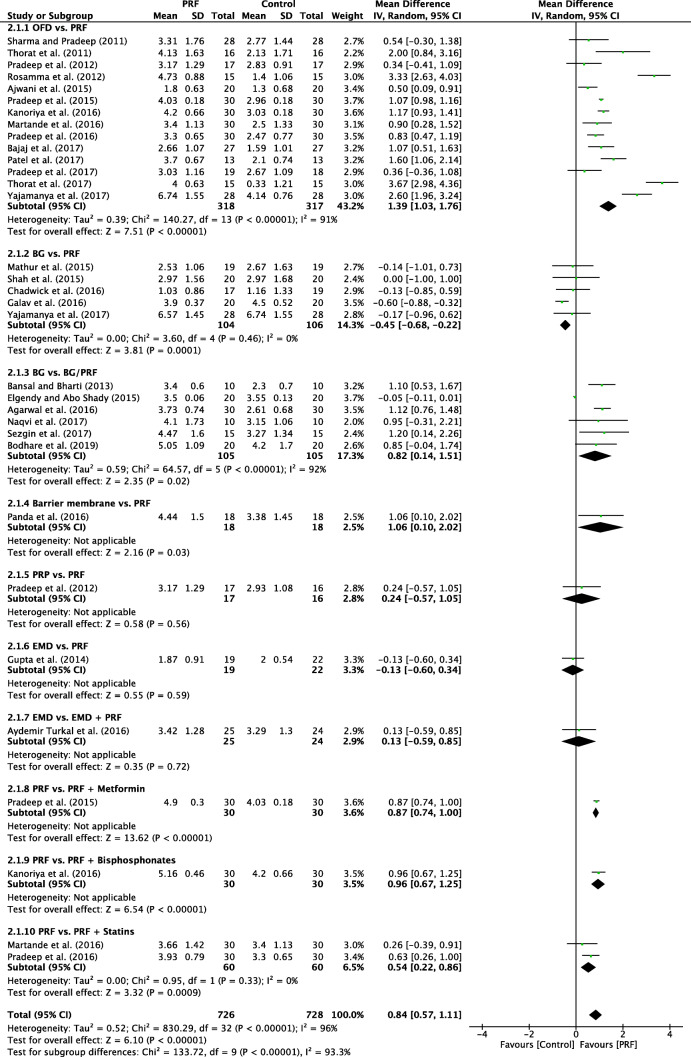
Forest plot for the event “clinical attachment level” (reported in mm) for intrabony defects

For CAL, the random-effects model was used due to the moderate heterogeneity among the analyzed subgroups (*P* < 0.00001; *I*^2^ = 96%). One subgroup showed a significant difference (*P* < 0.0001) in favor of autogenous graft when compared to PRF, with a mD of − 0.45 (95% CI − 0.68 to − 0.22). However, in the overall effect there was a significant difference (*P* < 0.00001) in favor of the PRF group when compared to the control group, with mD of 0.84 (95% CI 0.57 to 1.11) (Fig. 4).

#### Radiographic bone fill

**Fig. 5:**
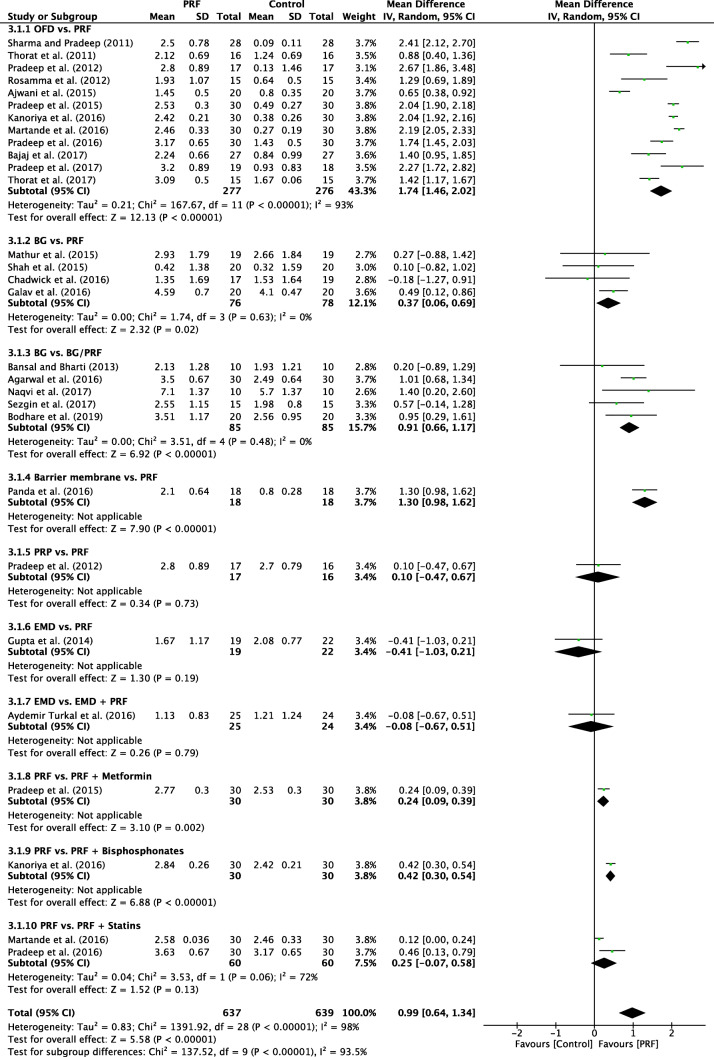
Forest plot for the event “bone fill” (reported in % change) for intrabony defects

A random-effects model was used to evaluate the RBF due to the high heterogeneity that was found between the subgroups (*P* < 0.00001; *I*^2^ = 98%). No subgroup showed a significant result in favor of the control groups when compared to the PRF group. In overall effect, the use of PRF differed significantly (*P* = 0.11) in favor of PRF when compared to the control group, with a mD of 0.99 (95% CI 0.64 to 1.34) (Fig. 5).

### Assessment of the risk of bias

The overall risk of biased judgment of all studies was classified as “low risk of bias.” The RoB 2 analysis is shown in the Supplementary Appendix (S2).

## Discussion

The present SR and meta-analysis have investigated the use of PRF for reconstructive surgery in intrabony defects as evaluated in RCTs comparing it to all other treatment modalities. The aim was to more specifically address the use and recommendations for PRF for the treatment of periodontal two- and three-walled intrabony defects. Overall, the majority of studies to date compared the use of OFD/PRF versus OFD alone or OFD/BG versus OFD/PRF (Table 1). Furthermore, additional studies were gathered comparing OFD/EMD versus OFD/PRF, OFD/BM versus OFD/PRF, and OFD/PRF versus OFD/PRF/biomolecules. Below, we highlight and discuss the summary of evidence from the current categories and further discuss the strengths and limitations of each comparative analysis (Table 2).

### OFD alone versus with PRF

In total, 14 studies evaluated the use of PRF as an adjunct to OFD when compared to OFD alone (Table 1). In summary, 13 of the 14 studies demonstrated statistically significant clinical improvements in mean PD reduction—1.26 mm (Fig. 2), 11 of 14 studies demonstrated statistically significant improvements in mean CAL gain 1.39 mm (Fig. 4), and all studies showed statistically significant improvement in terms of bone fill (Fig. 5). In summary, it was observed that on average, the results from 14 RCTs demonstrated a statistically significant relative PD reduction of ~ 1.3 mm and ~ 1.5 mm CAL gain when PRF was additionally filled into intrabony defects following OFD (Figs. 2, 3, and 4).

### Bone graft versus PRF

In a second series of studies, five studies evaluated the use of a BG versus PRF (Table 1). In general, no statistically significant difference was found between the two groups. One study demonstrated statistically significantly better results for PRF [51] and one study demonstrated statistically significantly more favorable outcomes for BG [34]. The three remaining studies demonstrated no statistically significant differences between the groups. The meta-analysis also demonstrated no statistically significant differences in PD reduction, CAL gain, or RBF between the two groups. Therefore, the data indicate that PRF may lead to comparable clinical outcomes than those obtained with BG when used for intrabony defect repair/regeneration.

### Bone graft versus bone graft + PRF

In a third series of investigated studies, six studies evaluated the additional use of PRF to BG when compared to BG alone (Table 1). Of the six studies, two demonstrated a statistically significant improvement in PD and CAL gain when compared to BG alone [27, 52] while the other four studies demonstrated no statistically significant difference [31, 33, 39, 46]. A final reported ~ 0.5-mm non-significant reduction in PD was observed. When investigating CAL gain, two studies demonstrated a statistically significant advantage whereas three others have failed to demonstrate statistically significant improvements in CAL level (Fig. 4). Following meta-analysis, it was found that the additional use of PRF to BG led to a statistically significant ~ 1 mm gain in CAL when compared to BG alone and also statistically significant improvements in RBF. Therefore, some clinical benefit was observed when PRF was combined with a BG material. Potential reasons for these findings are likely multi-factorial. Many bone grafting materials, such as xenografts and the majority of synthetic materials, have no incorporation of extracellular matrix components or growth factors. Therefore, one hypothesized reason for the additional benefit of including PRF to a BG could be its new incorporation of regenerative cells and growth factors that contribute to the regenerative process. Previous in vitro research investigating PRF has demonstrated its ability to improve PDL cell migration, proliferation, and wound closure rates [55]. Furthermore, PRF also contains supra-physiological concentrations of defense-fighting leukocytes. Since periodontal pockets harbor a number of periodontal pathogens, it is possible that leukocytes may aid in the defense against potential bacterial contamination/invasion. Lastly, basic science studies have now demonstrated that PRF promotes an anti-inflammatory environment [56, 57]. Recent research has shown that PRF has the ability to favor M2 macrophage polarization and also decreases tissue inflammation [56, 57]. It also possesses some anti-bacterial/antimicrobial activity, thereby favoring potential wound healing of periodontal pockets [58, 59]. Taken together, each of the aforementioned parameters is thought to at least in part contribute toward periodontal regeneration when PRF is utilized in combination with a BG.

### Additional randomized clinical studies evaluating PRF

An additional eight studies evaluated the use of PRF in various RCTs as highlighted herein. No meta-analysis could be performed but general trends were reported (Figs. 2, 3, 4, and 5) [29, 35, 37, 40, 43–45]. The comparison investigating PRF versus a collagen barrier membrane yielded no statistically significant difference in terms of PD reduction. However, statistically significant improvements were observed for CAL and RBF favoring the PRF group when compared to collagen membranes [40]. Interestingly, no differences in any of the investigated parameters were found for single RCTs investigating (1) PRP versus PRF [45], (2) EMD versus PRF [35], or (3) EMD versus EMD + PRF [29].

Lastly, four studies have investigated PRF in combination with either (1) metformin [44], (2) bisphosphonates [36] or (3) statins [37, 43]. There was a statistically significant advantage in PD reduction, CAL gain, and RBF for the combined use of PRF with each of the above modalities when compared to utilizing PRF alone. Although few studies have thus far characterized their potential benefit, these relatively novel findings support the more recent trends favoring more “personalized” medicine as regenerative strategies. Thus, future research investigating specific patient populations (e.g., osteoporotic women) may potentially and more specifically target the local use of additional biomolecules (such as bisphosphonates) favoring more specific bioactivity (anti-resorptive properties) favoring a more personalized treatment protocol. Furthermore, the use of antibiotic therapy in certain patients with aggressive periodontitis may benefit from more “personalized” antibiotic therapy. Since PRF may be utilized as a three-dimensional matrix with long-term delivery of small biomolecules, PRF may therefore be utilized as a therapeutic drug delivery system as previously reported [60]. Nevertheless, it remains somewhat unclear the modes of action of certain strategies such as additional combination of metformin to PRF. Future research investigating PRF as a potential drug delivery system for various local therapeutic agents/biomolecules with their better understanding may provide further clinical benefit. At present, however, the above trends are simply reported in single RCTs with much further research needed on the topic.

### Implications for clinical practice and future direction

The latest guidelines from the EFP determined in a study titled “Regenerative surgery versus access flap for the treatment of intra-bony periodontal defects: a systematic review and meta-analysis” that EMD or GTR in combination with papillary preservation flaps should be considered the treatment of choice for residual pockets with deep (≥ 3 mm) intrabony defects [61]. With the fact that blood clot seems to be a very important aspect for periodontal regeneration, it must first be noted that there remains a great need for human/animal histology with lots of effort to deliver studies that compare standard periodontal regenerative procedures with PRF.

Despite the fact that PRF is only beginning to be more commonly utilized in routine clinical practice for the treatment of intrabony defects, it remains interesting to note that 27 RCTs have thus far evaluated its potential for periodontal regeneration/repair. The formation of a blood clot alone has been shown to be one of the key necessary features in order for periodontal regeneration to take place, as long as bacterial pathogens have been completely eliminated. Evidence from the literature suggests that blood clot formation alone is enough to treat a number of intrabony defects where space maintenance is not an issue (two- or three-walled defects) [62]. PRF therefore acts in a similar fashion whereby the fibrin scaffold can be inserted into the periodontal pocket acting as a stable clot, with significant increases in platelets, leukocytes, and growth factors. While periodontal regeneration remains complex due to the number of tissues needed to be regenerated (new cementum, periodontal ligament, and alveolar bone), as well as the fact that Sharpey’s fibers need to be oriented functionally to support the tooth apparatus, it remains difficult to assess whether PRF actually leads to true periodontal regeneration since no human histological evidence exists to date on the topic (despite the nearly 30 RCTs having been performed). Nevertheless, it is known that periodontal disease is caused by bacterial pathogens and an increase in regenerative growth factors and cells, as well as its incorporation of defense-fighting leukocytes is certainly hypothesized to favor defect resolution and potentially mitigate tissue inflammation. Furthermore, angiogenesis is an important factor for tissue regeneration, and PRF releases a number of pro-angiogenic and pro-fibrotic agents capable of further speeding periodontal tissue repopulation [9, 62].

The biological advantages of PRF have been shown to act locally by quickly stimulating a large number of cell types by influencing their recruitment, proliferation and/or differentiation. These have previously been shown to include endothelial cells, gingival fibroblasts, chondrocytes, and osteoblasts, thereby having the potential effect to act locally and affect various cell types [63, 64]. Thus, PRF may prove beneficial for the regeneration of specific tissues such as the periodontium since several cell types and tissue types are required to regenerate in order for periodontal regeneration to occur. While it is known that beneficial effects of PRF may partially be due to the large number of secreted autologous blood-derived growth factors, it remains interesting to point out the fact that rhPDGF (which is approved by the FDA) has been one of the main recombinant growth factors sold to date in North America for the regeneration of periodontal tissues [65–68]. Although recombinant proteins have a regenerative potential well documented in the literature [69–71], their associated costs and other secondary adverse effects, including biocompatibility, lower stability, and potential swelling, may favor the use of autologous PRF [72, 73]. Future comparative studies including a cost–benefit analysis between both modalities remain necessary.

It is also noted that of a total of 551 papers that were screened and 27 RCTs that met the inclusion criteria, studies were classified and presented into 10 categories, via comparison of clinical outcomes between PRF and other various treatment modalities/approaches. It remains interesting to point out that of the studies, three major groups of studies were gathered as follows: (1) therapeutic modalities with or without PRF (OFD vs. OFD/PRF, OFD/BG vs. OFD/BG/PRF, and OFD/EMD vs. OFD/EMD/PRF); (2) treatment modalities in comparison to PRF (OFD/PRF vs. OFD/BG, OFD/BM, OFD/PRP, and OFD/EMD, respectively); (3) therapeutic modalities in comparison of OFD/PRF with additional small biomolecules (OFD/PRF vs. OFD/PRF/metformin, OFD/PRF/bisphosphonates, and OFD/PRF/statins, respectively).

All therapeutic modalities with addition of PRF to their surgical approach (group 1) demonstrated better outcomes apart from one study comparing OFD/EMD versus OFD/EMD/PRF. Each of the treatment modalities comparing PRF alone to other regenerative strategies (group 2) found similar clinical outcomes between both groups. Each of the therapeutic modalities utilizing PRF with addition of small biomolecules (group 3) found improved clinical outcomes with the addition of either metformin, bisphosphonates, or statins. Most of the studies dealt with the therapeutic modalities with or without PRF (group 1; *n* = 21, 78%). It thus remains interesting to note that generally speaking, the additional use of PRF tends to favor regenerative outcomes of IBDs, and addition of small biomolecules may further improve such outcomes. Future research investigating more precisely when PRF should be utilized in combination approaches versus as a sole regenerative modality needs further clarification.

Several research topics also remain at the forefront of needed research in this space. As previously mentioned, it remains interesting to point out that no single study has characterized PRF at the histological level in a well-characterized human study. It has already been well established in the literature that PRF favors soft tissue wound healing when compared to hard tissues [74]. Since periodontitis is not only characterized by PDL breakdown but also that of cementum and alveolar bone, the regenerative potential of each of these tissues needs to be further characterized via histological evaluation, ideally in human studies. Furthermore, it remains astonishing that very few included trials addressed with their research these valid questions: (1) collagen membranes without hard substitute support, (2) bone grafting of any kind of material without barriers, (3) PRF as a substitute for collagen membranes, etc. The next wave of research should more specifically address these important topics to better understand the additional role/benefit PRF may serve under such conditions. Furthermore, an array of different surgical procedures utilized to treat IBDs exists. Previously, it was shown that great variability in surgical approaches was discussed when PRF was used for the treatment of gingival recessions [75]. Future research investigating more precisely various surgical difference such as flap design and surgical techniques (e.g., MIST and M-MIST) should be further evaluated in future studies to better determine optimal surgical approaches when using PRF in regenerative therapy of IBDs.

## Conclusion

The data from the present SR with meta-analysis demonstrate that OFD/PRF leads to statistically significant clinical improvements in PD reduction, CAL gain, and RBF when compared to OFD alone. Furthermore, the data suggest that comparable results can be obtained when intrabony defects are filled with either PRF or a BG and statistically significant improvements in CAL and RBF were observed when PRF was combined with BG. Future research may be warranted to evaluate the use of PRF in combination with various additional small biomolecules such as metformin, bisphosphonates, statins, and/or antibiotics to additionally improve the clinical outcomes. In addition, animal and human histological evidence is needed to verify if PRF actually leads to true periodontal regeneration.

## Supplementary Information

ESM 1 (DOCX 24 kb)
