# Supplementary material for: Use of platelet-rich fibrin for the treatment of periodontal intrabony defects: a systematic review and meta-analysis
Source: Clin Oral Investig. 2021 Feb 20;25(5):2461–78. doi: 10.1007/s00784-021-03825-8 (PMC8060184; doi:10.1007/s00784-021-03825-8)
Supplement: Supplementary file 1 — (DOCX 24 kb) [file 784_2021_3825_MOESM1_ESM.docx]

**S1**

| **Databases** | **Keywords** |
| --- | --- |
| PubMed | #1 alveolar bone loss[MeSH Terms] OR guided tissue regeneration[MeSH Terms] OR intrabony defects[Title/Abstract] OR bone defects[Title/Abstract] OR infrabony defects[Title/Abstract] OR guided tissue regeneration[Title/Abstract] OR #2 graft material[Title/Abstract] OR blood buffy coat[MeSH Terms] OR leucocyte platelet-rich fibrin[Title/Abstract] OR platelet-rich fibrin[Title/Abstract] OR L-PRF[Title/Abstract] OR PRF[Title/Abstract]  #1 AND #2 |
| Scopus | #1 “alveolar bone loss” OR “guided tissue regeneration” OR “intrabony defects” OR “bone defects” OR “infrabony defects” OR “guided tissue regeneration” OR #2 “graft material” OR “blood buffy coat” OR “leucocyte platelet-rich fibrin” OR “platelet-rich fibrin” OR “L-PRF” OR “PRF”  #1 AND #2 |
| Cochrane Library (CENTRAL) | #1 alveolar bone loss OR guided tissue regeneration OR intrabony defects OR bone defects OR infrabony defects OR guided tissue regeneration OR #2 graft material OR blood buffy coat OR leucocyte platelet-rich fibrin OR platelet-rich fibrin OR L-PRF OR PRF  #1 AND #2 |
| Lilacs | #1 “alveolar bone loss” OR “guided tissue regeneration” OR “intrabony defects” OR “bone defects” OR “infrabony defects” OR “guided tissue regeneration” OR #2 “graft material” OR “blood buffy coat” OR “leucocyte platelet-rich fibrin” OR “platelet-rich fibrin” OR “L-PRF” OR “PRF”  #1 AND #2 |
| Grey Literature Report  OpenGrey | #1 alveolar bone loss OR guided tissue regeneration OR intrabony defects OR bone defects OR infrabony defects OR guided tissue regeneration OR #2 graft material OR blood buffy coat OR leucocyte platelet-rich fibrin OR platelet-rich fibrin OR L-PRF OR PRF  #1 AND #2 |

**S2**

| **Domains/Author/Year** | **Sharma and Pradeep (2011)** | **Thorat et al. (2011)** | **Rosamma et al. (2012)** | **Pradeep et al. (2012)** | **Bansal and Bharti (2013)** | **Gupta et al. (2014)** |
| --- | --- | --- | --- | --- | --- | --- |
| **Bias arising from the randomization process** | Low | Low | Low | Low | Low | Low |
| **Bias due to deviations from intended interventions** | Low | Low | Low | Low | Low | Low |
| Bias due to missing outcome data | Low | Low | Low | Low | Low | Low |
| Bias in measurement of the outcome | Low | Low | Low | Low | Low | Low |
| Bias in selection of the reported result | Low | Low | Low | Low | Low | Low |
| **Overall risk of bias judgment** | **Low Risk** | **Low Risk** | **Low Risk** | **Low Risk** | **Low Risk** | **Low Risk** |

| **Domains/Author/Year** | **Ajwani et al. (2015)** | **Mathur et al. (2015)** | **Shah et al. (2015)** | **Elgendy and Abo Shady (2015)** | **Pradeep et al. (2015)** | **Galav et al. (2016)** |
| --- | --- | --- | --- | --- | --- | --- |
| **Bias arising from the randomization process** | Low | Low | Low | Low | Low | Low |
| **Bias due to deviations from intended interventions** | Low | Low | Low | Low | Low | Low |
| Bias due to missing outcome data | Low | Low | Low | Low | Low | Low |
| Bias in measurement of the outcome | Low | Low | Low | Low | Low | Low |
| Bias in selection of the reported result | Low | Low | Low | Low | Low | Low |
| **Overall risk of bias judgment** | **Low Risk** | **Low Risk** | **Low Risk** | **Low Risk** | **Low Risk** | **Low Risk** |

| **Domains/Author/Year** | **Chadwick et al. (2016)** | **Agarwal et al. (2016)** | **Panda et al. (2016)** | **Kanoriya et al. 2016** | **Martande et al. (2016)** | **Pradeep et al. (2016)** |
| --- | --- | --- | --- | --- | --- | --- |
| **Bias arising from the randomization process** | Low | Low | Low | Low | Low | Low |
| **Bias due to deviations from intended interventions** | Low | Low | Low | Low | Low | Low |
| Bias due to missing outcome data | Low | Low | Low | Low | Low | Low |
| Bias in measurement of the outcome | Low | Low | Low | Low | Low | Low |
| Bias in selection of the reported result | Low | Low | Low | Low | Low | Low |
| **Overall risk of bias judgment** | **Low Risk** | **Low Risk** | **Low Risk** | **Low Risk** | **Low Risk** | **Low Risk** |

| **Domains/Author/Year** | **Turkal et al. (2016)** | **Bajaj et al. (2017)** | **Patel et al. (2017)** | **Pradeep et al. (2017)** | **Thorat et al. (2017)** | **Sezgin et al. (2017)** |
| --- | --- | --- | --- | --- | --- | --- |
| **Bias arising from the randomization process** | Low | Low | Low | Low | Low | Low |
| **Bias due to deviations from intended interventions** | Low | Low | Low | Low | Low | Low |
| Bias due to missing outcome data | Low | Low | Low | Low | Low | Low |
| Bias in measurement of the outcome | Low | Low | Low | Low | Low | Low |
| Bias in selection of the reported result | Low | Low | Low | Low | Low | Low |
| **Overall risk of bias judgment** | **Low Risk** | **Low Risk** | **Low Risk** | **Low Risk** | **Low Risk** | **Low Risk** |

| **Domains/Author/Year** | **Yajamanya et al. (2017)** | **Naqvi et al. (2017)** | **Bodhare et al. (2019)** |
| --- | --- | --- | --- |
| **Bias arising from the randomization process** | Low | Low | Low |
| **Bias due to deviations from intended interventions** | Low | Low | Low |
| Bias due to missing outcome data | Low | Low | Low |
| Bias in measurement of the outcome | Low | Low | Low |
| Bias in selection of the reported result | Low | Low | Low |
| **Overall risk of bias judgment** | **Low Risk** | **Low Risk** | **Low Risk** |
